# Pharmaceutical Drug Metformin and MCL1 Inhibitor S63845 Exhibit Anticancer Activity in Myeloid Leukemia Cells via Redox Remodeling

**DOI:** 10.3390/molecules26082303

**Published:** 2021-04-15

**Authors:** Giedrė Valiulienė, Aida Vitkevičienė, Giedrė Skliutė, Veronika Borutinskaitė, Rūta Navakauskienė

**Affiliations:** Department of Molecular Cell Biology, Institute of Biochemistry, Life Sciences Center, Vilnius University, Sauletekio av. 7, LT-01257 Vilnius, Lithuania; aida.vitkeviciene@gmc.vu.lt (A.V.); giedre.skliute@gmc.vu.lt (G.S.); veronika.borutinskaite@bchi.vu.lt (V.B.); ruta.navakauskiene@bchi.vu.lt (R.N.)

**Keywords:** acute myeloid leukemia (AML), metformin, MCL-1 inhibitor S63845, reactive oxygen species (ROS)

## Abstract

Metabolic landscape and sensitivity to apoptosis induction play a crucial role in acute myeloid leukemia (AML) resistance. Therefore, we investigated the effect of metformin, a medication that also acts as an inhibitor of oxidative phosphorylation (OXPHOS), and MCL-1 inhibitor S63845 in AML cell lines NB4, KG1 and chemoresistant KG1A cells. The impact of compounds was evaluated using fluorescence-based metabolic flux analysis, assessment of mitochondrial Δψ and cellular ROS, trypan blue exclusion, Annexin V-PI and XTT tests for cell death and cytotoxicity estimations, also RT-qPCR and Western blot for gene and protein expression. Treatment with metformin resulted in significant downregulation of OXPHOS; however, increase in glycolysis was observed in NB4 and KG1A cells. In contrast, treatment with S63845 slightly increased the rate of OXPHOS in KG1 and KG1A cells, although it profoundly diminished the rate of glycolysis. Generally, combined treatment had stronger inhibitory effects on cellular metabolism and ATP levels. Furthermore, results revealed that treatment with metformin, S63845 and their combinations induced apoptosis in AML cells. In addition, level of apoptotic cell death correlated with cellular ROS induction, as well as with downregulation of tumor suppressor protein MYC. In summary, we show that modulation of redox-stress could have a potential anticancer activity in AML cells.

## 1. Introduction

Acute myeloid leukemia (AML) is one of the most common forms of blood cancer in adults. Typically, the five-year survival rate for AML patients is ~29% [1]. The prognosis of specific AML cases varies widely as patient age and type of AML should be taken into account. For example, APL (acute promyelocytic leukemia) subtype of AML is mostly well managed, while others have a more complicated prognosis, as AML resistance and relapse still remain very serious issues [2]. Therefore, better treatment options in AML cases remain an urgent need.

A very interesting and innovative approach is a drug repositioning—when existing drugs are investigated for new therapeutic purposes. For example, metformin (*N*,*N*-dimethylbiguanide), which is applied widely to treat the symptoms of hyperglycemia in type 2 diabetes [3] and also used in the treatment of polycystic ovary syndrome [4], may be repurposed to manage cancer as well. Metformin was shown to have a cytostatic activity; it can strengthen chemotherapeutic effect [5]. In human breast cancer and osteosarcoma cells metformin was shown to induce the generation of reactive oxygen species (ROS) and partly elicit its anticancerous effect via redox remodeling [6,7]. Regarding leukemia research, metformin was demonstrated to sensitize AML cell lines to cytarabine [8]. Recently, we demonstrated that metformin in combination with cytarabine, and venetoclax also slightly inhibited AML patients’ cell proliferation and profoundly reduced oxidative phosphorylation (OXPHOS) rate ex vivo [9]. It has been reported consistently that metformin elicits its effect on oxidative phosphorylation via inhibition of Complex I [10].

As mitochondrial oxidative phosphorylation is an essential factor in AML chemoresistance [11], targeting mitochondrial respiration combined with modulation of other cancer-associated pathways may be beneficial in AML treatment. Since the survival of cancer cells is heavily promoted by anti-apoptotic BCL-2 family members, such as BCL-2 (B cell lymphoma-2) and MCL-1 (myeloid cell leukemia-1), which prevent malignant cells from the mitochondrial cell death pathway [12], targeting both mitochondrial respiration and intrinsic apoptotic processes may suggest more potent therapeutic strategies. A recent study by Ewald et al. [13] revealed that MCL-1 may be a more universal therapeutic target in AML compared to BCL-2. S63845 (α(R)-[[(5S)-5-[3-chloro-2-methyl-4-[2-(4-methyl-1-piperazinyl)ethoxy]phenyl]-6-(5-fluoro-2-furanyl)thieno[2,3-d]pyrimidin-4-yl]oxy]-2-[[1-(2,2,2-trifluoroethyl)-1H-pyrazol-5-yl]methoxy]-benzenepropanoic acid) is an inhibitor of MCL-1, a pro-survival protein that is highly overexpressed in many cancer types and in AML as well [14,15]. Research has demonstrated that S63845 induced cell death in various AML cells [13]. In addition, AML cells that are in general resistant to venetoclax have been shown to be very sensitive to S63845 [16].

In the present study we aimed to test the effect of antidiabetic drug metformin and MCL-1 inhibitor S63845 in AML cell lines NB4 (APL subtype), KG1 and chemoresistant AML cell line KG1A. We were mainly interested in the potential of metabolic and redox modulation, as well as an effect on growth restriction. Therefore, we treated cells with metformin and S63845 alone or in combination. It should be emphasized that in this experimental set-up the supratherapeutic doses of metformin (10 mM) were used in order to alter the mitochondrial metabolism. After the treatments, we evaluated changes in cell proliferation and viability, metabolic activity, as well as ROS production and induction of apoptotic cell death.

## 2. Results

### 2.1. Metformin and S63845 Cytotoxicity and Effect on AML Cell Proliferation and Viability

**Figure 1:**
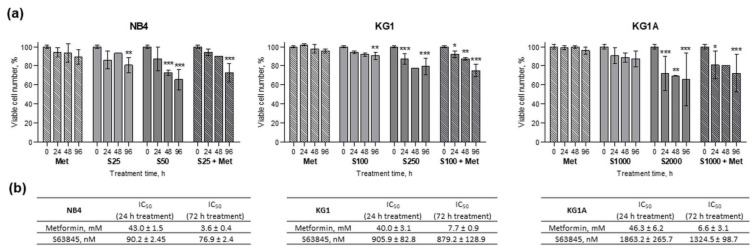
Effect of metformin and MCL-1 inhibitor S63845 on myeloid leukemia cell viability and induced cytotoxicity. (**a**) NB4, KG1 and KG1A cells were treated with 10 mM metformin and with different concentrations of MCL-1 inhibitor S63845 (S25–25 nM of S63845, etc.). Cell survival was evaluated by trypan blue exclusion test. Viability data were normalized to untreated controls. Results are mean ± S.D. (*n* ≥ 3). (**b**) Antiproliferative IC_50_ values for metformin and S63845 were evaluated using XTT Cell Proliferation Assay Kit (ATTC). Average ± S.D. is presented (*n* ≥ 3, except where columns are without error bars *n* = 1). Note: * denotes significant difference between treated vs. control cells with *p* < 0.05, ** denotes significant difference with *p* < 0.01, and *** denotes significant difference with *p* < 0.005, as evaluated using 1-way ANOVA with Dunnett post hoc test.

After AML cell treatment with antidiabetic drug and oxidative phosphorylation inhibitor metformin at 10 mM concentration and treatment with various concentrations of MCL-1 inhibitor S63845 (25–2000 nM), as well as after their combined treatments, cell viability (Figure 1a) and proliferation (see Figure S1 in Appendix No. 1, Supplementary Materials) were assessed using the trypan blue exclusion test. Agent concentrations were chosen after testing a range of different concentrations (data not shown). We selected effective cell proliferation and survival-inhibiting concentrations, which did not show too high toxicity. In general, NB4, KG1 and KG1A cells were sensitive to applied treatments, as reduction in cell proliferation and cell viability was observed. Effect of 10 mM metformin was of comparable degree between all cell lines, though metformin had a slightly stronger effect on cell viability in NB4 cells compared to KG1A cells. However, evidently NB4, KG1 and KG1A cells were not equally sensitive to MCL-1 inhibitor S63845. For example, inhibition of cell proliferation (Figure S1 in Appendix No. 1, Supplementary Materials) in NB4 cells was achieved when using 25 nM of S63845. In order to reach a similar effect in KG1 cells we had to use 100 nM of S63845, whereas in KG1A cells only 1000 nM of S63845 could slow down the cell proliferation. Using XTT Cell Proliferation Assay Kit (ATTC) we also determined the antiproliferative IC_50_ values for metformin and S63845 (Figure 1b), which confirmed that metformin did have very similar cytotoxicity patterns in NB4, KG1 and KG1A cells (e.g., after 24 h treatments IC_50_ values ranged 40.0–46.3 mM). IC_50_ analysis results also verified that NB4 cells were the most sensitive for S63845 treatment, whereas for KG1A cells approx. 20-fold higher doses were needed to reach IC_50_ (after 24 h treatments IC_50_ was 90.2 nM and 1863.2 nM in NB4 and KG1A cells, respectively).

All around, it should be stressed that in NB4, KG1 and KG1A cells co-treatments with S63845 and metformin possibly had an additive effect on cell viability, as combined treatments reduced viable cell numbers more efficiently than S63845 and metformin acting alone. However, further analysis is needed in order to find the combination index (CI) values and confirm or reject this hypothesis.

### 2.2. Energetic Profile Regulation by Metformin and S63845 in AML Cells

**Figure 2:**
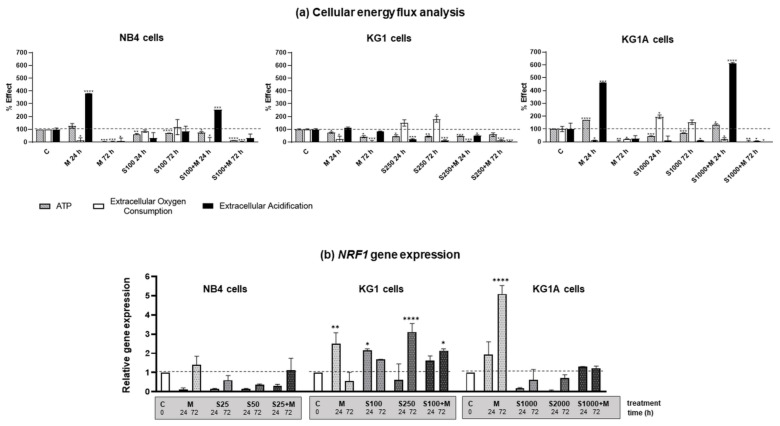
Cellular energy flux after treatment with metformin and MCL-1 inhibitor S63845. (**a**) Cellular energy flux for NB4 cells (treated with 100 nM S63845, 10 mM metformin and combination of 100 nM S63845 + 10 mM metformin), KG1 cells (treated with 250 nM S63845, 10 mM metformin and combination of 250 nM S63845 + 10 mM metformin), as well as KG1A cells (treated with 1000 nM S63845, 10 mM metformin and combination of 1000 nM S63845 + 10 mM metformin) shown as a percentage relative to untreated control cells. Comparative measurements after 24 and 72 h of incubation were taken with Extracellular Oxygen Consumption Assay (ab197243; seeded at 500,000 viable cells per well; white columns), Glycolysis Assay [Extracellular acidification] (ab197244; seeded at 500,000 viable cells per well; black columns) and Luminescent ATP Detection Assay Kit (ab113849; seeded at 75,000 viable cells per well; striped columns). (**b**) Gene expression changes of nuclear respiratory factor 1 (NRF1) after treatments were measured using RT-qPCR ΔΔCt method. HPRT1 gene expression was used for normalization; results are presented as relative changes in comparison to untreated cells; results are mean ± S.D. (*n* = 3). Note: * denotes significant difference between treated vs. control cells with *p* < 0.05, ** denotes significant difference with *p* < 0.01, *** denotes significant difference with *p* < 0.005 and **** denotes significant difference with *p* < 0.0001, as evaluated using 1-way ANOVA with Dunnett post hoc test.

Multiparametric analysis of cellular energy flux was performed using Extracellular Oxygen Consumption Assay together with Glycolysis Assay and Luminescent ATP Detection Assay (Figure 2a). A shift in the metabolic phenotype of NB4 cells (treated with 100 nM S63845, 10 mM metformin and combination of 100 nM S63845 + 10 mM metformin), KG1 cells (treated with 250 nM S63845, 10 mM metformin and combination of 250 nM S63845 + 10 mM metformin), as well as KG1A cells (treated with 1000 nM S63845, 10 mM metformin and combination of 1000 nM S63845 + 10 mM metformin) was evaluated upon 24- and 72-h-long treatments. As visible from the data, in all cell lines, treatment with metformin completely downregulated oxidative phosphorylation, as illustrated by the decrease in extracellular oxygen consumption rates by 91–97% (after 72 h treatment). It should be noticed that in NB4 and KG1A cells glycolysis rates were strongly boosted (up to 400–500% compared to control) after 24 h treatment with metformin. Accordingly, at this time point after treatment with metformin, no decline in ATP levels was registered in NB4 and KG1A cells. Related effects were also observed in NB4 and KG1A cells after 24-h-long combined treatments. However, after 72 h with metformin, as well as with the combined treatment, both NB4 and KG1A cells dropped in the levels of glycolysis rate as well as ATP quantity. In contrast to metformin, S63845, acting as a single agent in all cell lines that were tested, especially in KG1 and KG1A cells, diminished glycolysis, although it had no effect on oxidative phosphorylation rate or even slightly increased it. All in all, results of cellular energy flux analysis revealed that combined treatment with metformin and S63845, in comparison to metformin and S63845 acting alone, had a stronger inhibitory effect on AML cell oxidative phosphorylation and glycolysis rate and, consequently, on cellular ATP levels. The most robust response was registered in KG1A cells after 72 h exposure.

In addition, we investigated the effect that treatments had on the expression of nuclear respirator factor 1 (NRF1) gene, which is a well-known transcription factor regulating oxidative phosphorylation, mitochondrial biogenesis and response to oxidative stress [17]. In general, results of RT-qPCR analysis demonstrated that treatments with MCL-1 inhibitor S63845 diminished NRF1 gene expression in NB4 and KG1A cells, whereas in KG1 cells upregulation of NRF1 gene expression was registered (Figure 2b). Tendency of metformin to induce overexpression of NRF1 gene was also observed, particularly in KG1 and KG1A cells, whereas combined treatments had an intermediate effect.

### 2.3. Effect of Metformin and S63845 on AML Cell Redox Modulation

**Figure 3:**
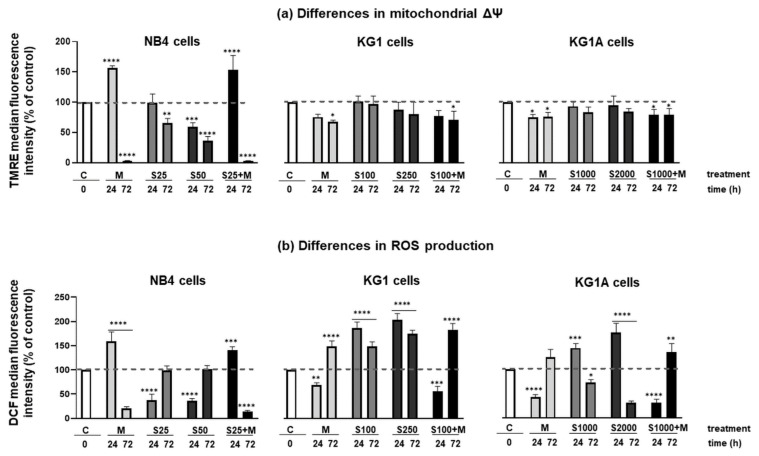
Effect of metformin and MCL-1 inhibitor S63845 on myeloid leukemia cell mitochondrial membrane potential and cellular reactive oxygen species production. For subsequent flow cytometric analysis NB4 cells were treated with 25 nM and 50 nM S63845, 10 mM metformin and combination of 25 nM S63845 + 10 mM metformin. KG1 cells were treated with 100 nM and 250 nM S63845, 10 mM metformin and combination of 100 nM S63845 + 10 mM metformin. KG1A cells were treated with 1000 nM and 2000 nM S63845, 10 mM metformin and combination of 1000 nM S63845 + 10 mM metformin. (**a**) Mitochondrial membrane potential (Δψm) of control (untreated) and treated NB4, KG1 and KG1A cells was evaluated using TMRE Mitochondrial membrane potential assay kit (ab113852) after 24 and 72 h of incubation. (**b**) Measurements of ROS production in control (untreated) and treated NB4, KG1 and KG1A cells were performed using Abcam DCFDA Cellular ROS detection assay kit (ab113851) after 24 and 72 h of incubation. Note: * denotes significant difference between treated vs. control cells with *p* < 0.05, ** denotes significant difference with *p* < 0.01, *** denotes significant difference with *p* < 0.005 and **** denotes significant difference with *p* < 0.0001, as evaluated using 1-way ANOVA with Dunnett post hoc test.

Mitochondria have a major role in regulating cellular energetics, as well as activating the intrinsic pathway of controlled cell death. Therefore, we further evaluated metformin and S63845 activity on NB4, KG1 and KG1A cell mitochondrial potential and ROS production. Results of flow cytometric TMRE dye accumulation analysis (Figure 3a) revealed that in NB4 cells 24 h treatment with 10 mM metformin, as well as 24 h treatment with a combination of 25 nM S63845 + 10 mM metformin significantly increased Δψ (by approx. 60% compared to untreated control cells). However, opposite results were obtained in KG1 and KG1A cells, when metformin alone or together with S63845 reduced the mitochondrial membrane potential. It also should be stressed that MCL-1 inhibitor S63845, when used as a single agent, had no effect on Δψ. Nevertheless, after 72 h exposure both metformin alone and a combined treatment with S63845 diminished AML cell mitochondrial membrane potential.

**Figure 4:**
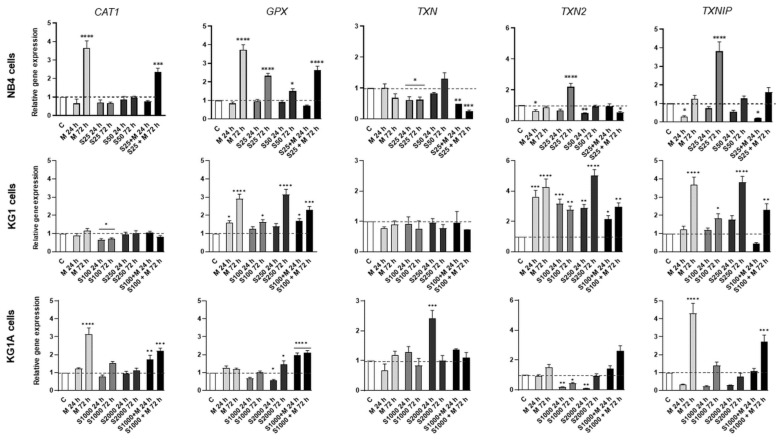
Metformin and MCL-1 inhibitor S63845 induced changes in the expression of antioxidant system genes. NB4 cells were treated with 25 nM and 50 nM S63845, 10 mM metformin and combination of 25 nM S63845 + 10 mM metformin. KG1 cells were treated with 100 nM and 250 nM S63845, 10 mM metformin and combination of 100 nM S63845 + 10 mM metformin. KG1A cells were treated with 1000 nM and 2000 nM S63845, 10 mM metformin and combination of 1000 nM S63845 + 10 mM metformin. Gene expression changes of antioxidant system genes *CAT1*, *GPX*, *TXN*, *TXN*2 and *TXNIP* after 24 and 72 h of treatment were measured using RT-qPCR ΔΔCt method. *HPRT1* gene expression was used for normalization; results are presented as relative changes in comparison to untreated cells; results are mean ± S.D. (*n* = 3). Note: * denotes significant difference between treated vs. control cells with *p* < 0.05, ** denotes significant difference with *p* < 0.01, *** denotes significant difference with *p* < 0.005 and **** denotes significant difference with *p* < 0.0001, as evaluated using 1-way ANOVA with Dunnett post hoc test.

As shown in Figure 3b, metformin increased intracellular ROS production in NB4 cells, whereas in KG1 and KG1A cells the highest increase in ROS production was registered upon treatments with S63845. Therefore, the effects of metformin and MCL-1 inhibitor S63845 on antioxidant enzymes were investigated (Figure 4). We hypothesized that upregulation of intracellular ROS levels would result from the downregulation of gene expression of antioxidant enzymes, such as catalase 1 (CAT1), glutathione peroxidase (GPX) and thioredoxin system enzymes. Results showed that CAT1 gene expression was reduced only in KG1 cells, when treated with 100 nM S63845 (24 and 72 h), whereas treatment with metformin significantly upregulated expression of CAT1 in NB4 and KG1A cells. MCL-1 inhibitor S63845 also reduced GPX gene expression in KG1A cells after 24 h treatment, though, in general, 72-h-long treatments with metformin, S63845 and their combinations, tended to upregulate GPX expression in tested AML cells. However, our further analysis demonstrated that MCL-1 inhibitor S63845 had a significant effect on thioredoxin system regulation: in NB4 cells thioredoxin-1 (TXN) was downregulated 1.6-fold after treatment with 25 nM S63845 (24 and 72 h). In addition, the effect was much stronger when 25 nM S63845 was applied in combination with 10 mM metformin (expression was downregulated 4-fold). Supporting our hypothesis, inhibition of mitochondrial thioredoxin (TXN2) gene expression was also observed in KG1A cells upon treatments with S63845. However, no reduction in gene expression of thioredoxin reductases 1 and 2 (TXNRD1 and TXNRD2) was detected, while after treatments their expression was evidently increased (see Figure S2 in Appendix No. 1, Supplementary Materials). Nonetheless, it should be noticed that gene expression of tumor suppressor thioredoxin interacting protein (TXNIP), which is a well-known inhibitor of thioredoxin-1 [18], was significantly upregulated after used treatments. For example, in NB4 cells gene expression of TXNIP was increased the most strongly after treatment with S63845, while in KG1A cells the most significant effect was obtained after treatment with metformin.

### 2.4. Treatment-Induced Apoptosis and Apoptosis-Related Gene and Protein Expression

**Figure 5:**
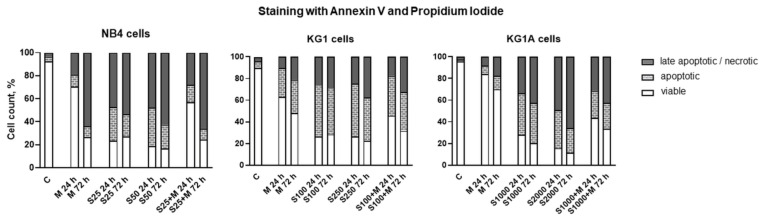
Metformin and MCL-1 inhibitor S63845 effect on cell apoptosis. Cell apoptosis of NB4 cells (treated with 25 nM and 50 nM S63845, 10 mM metformin and combination of 25 nM S63845 + 10 mM metformin), KG1 cells (treated with 100 nM and 250 nM S63845, 10 mM metformin and combination of 100 nM S63845 + 10 mM metformin), as well as KG1A cells (treated with 1000 nM and 2000 nM S63845, 10 mM metformin and combination of 1000 nM S63845 + 10 mM metformin) was evaluated by staining with Annexin V and Propidium Iodide after 24 and 72 h of treatment. Results are mean (*n* = 3; S.D. < ± 10%). Note: statistical analysis was performed using 2-way ANOVA with Tukey’s multiple comparison test (due to the complexity of data, exact statistical analysis results are presented in Appendix No. 2, (Supplementary Materials).

The type of cell death induced in AML cells by treatment with metformin, MCL-1 inhibitor S63845 or their combination was determined by staining with Annexin V and Propidium Iodide (Figure 5). Analysis revealed that NB4, KG1 and KG1A cells treated with metformin at 10 mM concentration and with S63845 at selected concentrations (25 and 50 nM in NB4 cells, 100 and 250 nM in KG1 cells, as well as 1000 and 2000 nM in KG1A cells) induced cell apoptosis. In accordance with cell viability estimations (Figure 1), Annexin V and PI analysis confirmed that addition of metformin weakens the effect of S63845, especially in KG1 and KG1A cells. However, this phenomenon was not statistically significant (see the exact statistical data in Appendix No. 2, Supplementary Materials). Interestingly, in KG1 and KG1A cells metformin upregulated gene expression of cyclin-dependent kinase inhibitor p21 (see Figure S3 in Appendix No. 1, Supplementary Materials), whereas, after treatment with combinations of metformin and S63845, such an effect on CDKN1A expression was weaker.

**Figure 6:**
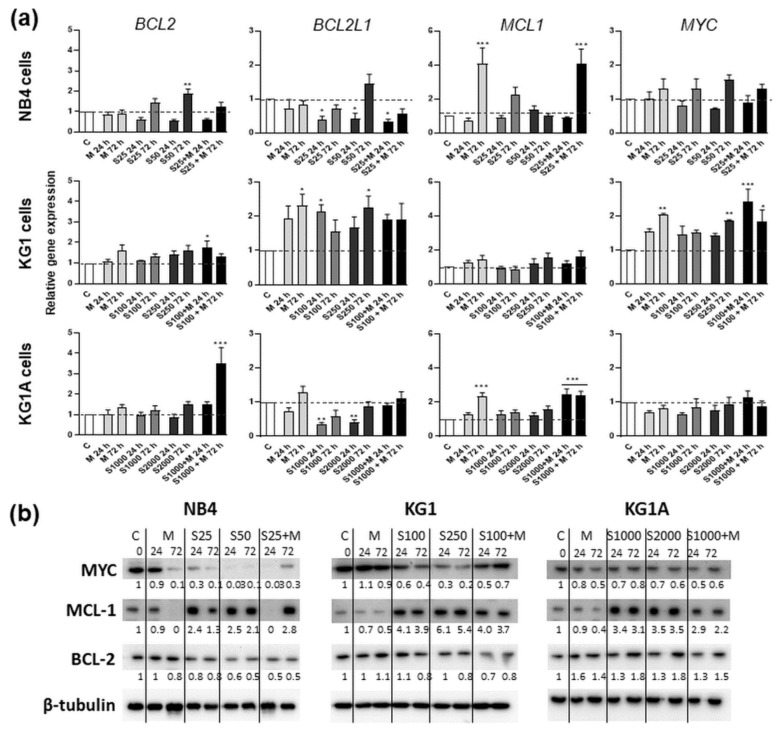
Anti-apoptotic gene and protein expression after treatment with metformin and MCL-1 inhibitor S63845. For subsequent analysis NB4 cells were treated with 25 nM and 50 nM S63845, 10 mM metformin and combination of 25 nM S63845 + 10 mM metformin. KG1 cells were treated with 100 nM and 250 nM S63845, 10 mM metformin and combination of 100 nM S63845 + 10 mM metformin. KG1A cells were treated with 1000 nM and 2000 nM S63845, 10 mM metformin and combination of 1000 nM S63845 + 10 mM metformin. C denotes control, untreated, cells. (**a**) Gene expression changes of apoptosis-suppressing genes *BCL2*, *BCL2L1*, *MCL1* and *MYC* after 24 and 72 h of treatment were measured using RT-qPCR ΔΔCt method. *HPRT1* gene expression was used for normalization; results are presented as relative changes in comparison to untreated cells; results are mean ± S.D. (*n* = 3). (**b**) Protein level changes after cell treatment with S63845 and metformin were assessed by immunoblot. β-tubulin was used as a loading control. Protein band intensity was measured using ImageJ software; band intensity results were normalized according to β-tubulin from the same membrane (*n* ≥ 2, representative results are displayed). Note: * denotes significant difference between treated vs. control cells with *p* < 0.05, ** denotes significant difference with *p* < 0.01, *** denotes significant difference with *p* < 0.005, as evaluated using 1-way ANOVA with Dunnett post hoc test.

To test whether metformin- and S63845-induced cell death was mediated by the regulation of oncogenic- and apoptosis-related factors that are linked to mitochondria, we further performed gene expression and Western blot analysis and measured the levels of BCL-2 (BCL-2), BCL-2-like-1 (BCL2L1), MCL-1 (MCL1) and MYC (MYC) (Figure 6a,b). Protein expression of MYC was downregulated time-dependently, especially strongly after incubation of cells with S63845 alone or in combination with metformin (in all cell lines that were tested, combined 24-h treatments with S63845 and metformin elicited stronger inhibitory effects on MYC protein levels compared to agents acting alone). However, we did not detect any significant decrease in MYC gene expression. Similarly, neither did we detect any significant downregulation of BCL2 and MCL1 gene expression. Nevertheless, BCL2L1 gene expression was significantly decreased in NB4 and KG1A cells after incubation with MCL-1 inhibitor S63845. Regarding protein level modulation, metformin had no effect or even lightly increased the levels of BCL-2, whereas treatment with S63845 had a reducing effect on NB4 and KG1 cells (however, in KG1A cells treatment with S63845 showed opposite results). It should be noted that in both NB4 and KG1 cells, combined treatments with S63845 and metformin had a stronger effect on BCL-2 protein level reduction than treatments with separate agents. Metformin also proved to be potent in MCL-1 level reduction, while treatment with S63845 evidently upregulated the levels of MCL-1. This indicated that S63845-induced apoptosis is not straightforwardly linked with the decrease in MCL-1 level, but rather with MCL-1 activity inhibition, as, in our study, increase in the levels of MCL-1 was concomitant with apoptotic cell death (Figure 5).

## 3. Discussion

Mitochondria-associated processes such as oxidative phosphorylation, production of reactive oxygen species and regulation of the intrinsic death pathway have a major role in the progression and survival of a plethora of cancer types, not excluding AML. Therefore, in this study we investigated the effect of the antidiabetic drug metformin, which is also an inhibitor of oxidative phosphorylation [19], alone and in combination with S63845, the inhibitor of anti-apoptotic protein MCL-1. We tested these agents on APL subtype cell line NB4, AML cell line KG1 and its chemoresistant counterpart KG1A cells.

We showed that metformin’s effect on cell proliferation was of comparable degree in all cell lines that were tested. However, differences were detected in cell viability, as 10 mM metformin decreased viable cell percentage the most strongly in NB4 cells, while the weakest impact was observed in chemoresistant KG1A cells (Figure 1a and Figure 5). In addition, downregulation of CDKN1A gene expression in NB4 cells was also registered after treatment with metformin, while in KG1 and KG1A cells metformin increased CDKN1A gene expression after prolonged exposure (Figure S3 in Appendix No. 1, Supplementary Materials). These observations coincide with cell viability and apoptosis evaluation data, as it is widely accepted that p21 can play the inhibitory role in apoptosis [20]. At this point it is very important to stress that co-treatment with metformin restrained S63845 effect on apoptosis induction, while, in general, S63845-induced level of apoptotic cell death in KG1 and KG1A cells was higher compared to the effect of metformin. However, results of cell viability analysis (Figure 1a) revealed that combined treatments with metformin and S63845 had a stronger effect, which was the most evident in KG1 and KG1A cells. Based on results obtained in our study, we hypothesize that apoptotic cell death of AML cells after treatments with either metformin, S63845 or their combination could be elicited, at least partly, via ROS level modulation. Previously it was thought that overproduction of ROS can only promote carcinogenesis and resistance to therapy, whereas currently it is assumed to have a split role—induction of ROS over the certain threshold can also lead to cancer cell death [21]. Our research demonstrated that in NB4 cells 10 mM metformin upregulated cellular ROS production by 1.6-fold, whereas in KG1A cells ROS accumulation was more pronounced upon treatments with S63845 (increased approx. 1.8-fold) (Figure 3b). Li et al. [7] showed that treatment with metformin can induce heightened ROS production in osteosarcoma cells. However, at the same time they did observe the reduction in mitochondrial membrane potential, while in our study, upregulation of ROS in NB4 cells after 24 h treatment was accompanied by the increase in Δψ. This seems credible, as, in general, increased mitochondrial membrane potential is associated with higher ROS production rates [22]. Of course, the mitochondrial pathway is not the only pathway that can generate ROS in myeloid leukemia cells, as NADPH oxidase is a critical enzyme in these cells, and its activation can lead to ROS accumulation and consequently it can potentiate apoptotic cell death [23]. It is plausible that MCL-1 inhibitor S63845 upregulated ROS generation in KG1 and KG1A cells mainly by acting via NADPH oxidase activity rather than through mitochondrial electron transport chain, as there were no significant changes in mitochondrial membrane potential detected (Figure 3a). However, we did notice a mild increase in the oxidative phosphorylation rate in KG1 and KG1A cells after treatments with S63845 (Figure 2a), which could be regarded as a compensatory mechanism due to reduction in glycolysis. The exact mechanism explaining the effect of S63845 on KG1 and KG1A cell glycolysis inhibition remains elusive and further studies are needed. Nevertheless, research by other authors has revealed that targeting MCL-1 indeed affects carbohydrate metabolism [24]. In addition, it should be emphasized that results of our study demonstrated that combined treatments, in comparison to metformin and S63845 acting alone, had more profound effects on inhibition of AML cell oxidative phosphorylation and glycolysis, as well as on reduction of ATP levels (Figure 2a).

Furthermore, in our study, treatment with MCL-1 inhibitor S63845 profoundly increased the levels of MCL-1 protein in all tested AML cells (Figure 6b). In addition, the increase of MCL-1 protein in cancer cells after treatment with S63845 has been demonstrated by other authors [15]. Their research revealed that binding of S63845 to MCL-1 disrupts MCL-1 interaction with pro-apoptotic proteins BAX/BAK, and thereafter indeed induces apoptosis. However, the increase in MCL-1 protein levels was associated with protein half-life extension [15]. In our study, increase in MCL-1 levels by treatment with S63845 was accompanied by the reduction in oncogenic protein MYC (Figure 6b). The strongest effect on MYC downregulation was observed in NB4 and KG1A cells, though in all cell lines that were tested, co-treatment with S63845 elevated the activity of metformin on MYC downregulation, as observed after 24 h combined treatments. Furthermore, co-treatments of NB4 and KG1 cells with S63845 and metformin had a stronger effect on BCL-2 protein level reduction than treatments with separate agents. These findings illustrated that the combination of MCL-1 inhibitor S63845 and metformin indeed has a repressive effect on NB4 and KG1 cells pro-survival proteins.

It is worth mentioning that in NB4 cells treatment with MCL-1 inhibitor S63845 statistically significantly lowered mitochondrial membrane potential and reduced production of cellular ROS (Figure 3a,b). Such a phenomenon could also be regarded as an apoptosis-promoting factor, as prolonged low values of Δψ are also threatening due to the cells’ inability to make enough ATP, as well as a reduced amount of ROS that could lead to the other kind of stress, the so-called “reductive stress” [25]. Therefore, drug-induced modulation of mitochondrial Δψ and ROS production could possibly play a major role in the anticancer activity of metformin and S63845 in AML cells. Obviously, we should admit that the concentration of metformin that was used in these experiments (10 mM) is too high for the clinical setting. Therefore, future studies will be necessary in order to test the potential of metformin and its combination with S63845 in AML treatment in vivo.

## 4. Materials and Methods

### 4.1. Cell Cultivation and Treatment

NB4 cell line (DSMZ, Braunschweig, Germany) was cultured in RPMI 1640 medium supplemented with 10% fetal bovine serum, 100 U/mL penicillin and 100 μg/mL streptomycin (Gibco, Carlsbad, CA, USA); KG1 and KG1A cell lines (ATCC, Manassas, VA, USA) were cultured in IMDM medium supplemented with 20% fetal bovine serum, 100 U/mL penicillin and 100 μg/mL streptomycin (Gibco, Carlsbad, CA, USA) at 37 °C in a humidified 5% CO_2_ atmosphere. For the treatment with metformin (Cayman Chemical Company, Ann Arbor, MI, USA) and S63845 (Cayman Chemical Company, Ann Arbor, MI, USA) cell seeding density was 0.5 × 10^6^ cells/mL. Cell proliferation and survival were assessed by trypan blue exclusion test using a hemocytometer. In short, 1 part of 0.2% trypan blue and 1 part of the cell suspension were mixed (100 µL of each). A drop of the mixture was applied to the hemocytometer (Neubauer-improved, 0.1 mm depth of chamber; Paul Marienfeld GmbH & Co. KG, Lauda-Königshofen, Germany) and manual counting using a binocular microscope was performed within 1 min. Unstained (viable) and stained (nonviable) cells were counted separately. Two technical replicates were performed. To obtain the total number of viable cells per ml of aliquot, the average count of viable cells from four hemocytometer fields (a square size 1 mm^2^) were multiplied by 2 (dilution factor) and once again multiplied by 10^4^. The percentage of viable cells was counted using the formula:

(1) $$\mathrm{viable}\;\mathrm{cells}\;\left(\%\right)=\frac{\mathrm{total}\;\mathrm{number}\;\mathrm{of}\;\mathrm{viable}\;\mathrm{cells}\;\mathrm{per}\;\mathrm{mL}\;\mathrm{of}\;\mathrm{aliquot}}{\mathrm{total}\;\mathrm{number}\;\mathrm{of}\;\mathrm{cells}\;\mathrm{per}\;\mathrm{mL}\;\mathrm{of}\;\mathrm{aliquot}}\times 100$$

Antiproliferative IC_50_ values were evaluated using XTT Cell Proliferation Assay Kit (ATTC, Manassas, VA, USA).

### 4.2. Apoptosis Evaluation

For the detection of apoptosis, ApoFlowEx^®^ FITC Kit (Exbio, Vestec, Czech Republic) was used. This assay detects viable, early apoptotic, and late apoptotic or necrotic cells depending on how cells are stained by Annexin V-FITC and Propidium Iodide. Stained cells were analyzed on the Millipore Guava^®^ easyCyte 8HT flow cytometer with InCyte 2.2.2 software.

### 4.3. Cellular Energy Flux Measurement

Cell extracellular oxygen consumption and extracellular acidification were measured using Extracellular Oxygen Consumption Assay Kit (Abcam, Cambridge, UK) and Glycolysis Assay Kit (Abcam, Cambridge, UK) according to the manufacturer’s instructions. Cells were treated with MCL1 inhibitor S63845 and metformin for 24 h and 72 h before measurements. Cells were harvested, washed, and seeded at 5 × 10^5^ viable cells/well in a 96-well plate, detection reagents were added and measurements performed in a plate reader. The aforementioned assays were validated using glucose oxidase as a positive control (Sigma-Aldrich, St. Louis, MO, USA). Cellular ATP concentration was evaluated using Luminescent ATP Detection Assay Kit (Abcam, Cambridge, UK) according to the manufacturer’s instructions. ATP standards provided in the kit were used in order to test the validity of the assay. Cells were seeded at 7.5 × 10^4^ viable cells/well in a white 96-well plate, detergent solution was added and incubated for 5 min to lyse cells and stabilize ATP, then substrate solution was added and the plate was stored in the dark until analysis of luminescence. For cell metabolic activity measurements the Thermo Scientific Varioskan^®^ plate reader (Thermo Fisher Scientific, Waltham, MA, USA) was used.

### 4.4. Assessment of Mitochondrial Membrane Potential

For TMRE Mitochondrial Membrane Potential Assay (Abcam, Cambridge, UK), 1 × 10^5^ of NB4, KG1 and KG1A cells were resuspended in PBS with 0.2% BSA and incubated with 400 nM TMRE for 30 min at 37 °C. Samples were analyzed with Millipore Guava^®^ easyCyte 8HT flow cytometer, using the InCyte 2.2.2 software. Ten thousand events were collected for each sample.

### 4.5. Cellular ROS Measurement

Levels of cellular reactive oxygen species (ROS) of NB4, KG1 and KG1A cells were determined using DCFDA Cellular ROS Detection Assay Kit (Abcam, Cambridge, UK). All procedures were carried out according to the manufacturer‘s instructions. In brief, 2.5 × 10^4^ cells/sample were incubated with 25 µM of 2′,7′–dichlorofluorescin diacetate (DCFDA) for 30 min at 37 °C and then analyzed using Millipore Guava^®^ easyCyte 8HT flow cytometer with InCyte 2.2.2 software. Ten thousand events were collected for each sample.

### 4.6. Gene Expression Analysis by RT-qPCR

Total RNA was purified using TRI Reagent (Zymo, Irvine, CA, USA). Traces of DNA in RNA preparations were removed using DNAse I, Amplification Grade (ThermoFisher Scientific, Waltham, MA, USA). cDNA was synthesized using SensiFAST™ cDNA Synthesis Kit (Bioline, Memphis, TN, USA) and qPCR was performed using SensiFAST™ SYBR^®^ No-ROX Kit (Bioline) on the RotorGene 6000 system (Corbett Life Science, QIAGEN, Hilden, Germany). Primer sequences (Metabion international AG, Planegg/Steinkirchen, Germany) are presented in Table S1 (see Table S1 in Appendix No. 1, Supplementary Materials). mRNA levels were normalized to HPRT1 expression. Relative gene expression was calculated using ΔΔCt method. Data are expressed as mean ±standard deviation (S.D.)

### 4.7. Immunoblotting

Cell lysates were prepared as described previously [26] and fractionated in 7.5–15% SDS-PAGE gradient electrophoresis gel. Proteins were transferred on PVDF membrane and specific proteins were detected using antibodies against BCL-2 (Cell Signalling Technology, Danvers, MA, USA), MCL-1 (Proteintech, Rosemont, IL, USA), MYC (Novus Biologicals, Centennial, CO, USA) and β-tubulin (Abcam, Cambridge, UK). β-tubulin was used as a loading control. Chemiluminescent detection was carried out using WesternBright ECL (Advansta, San Jose, CA, USA) on ChemiDoc™ XRS+ System (BIORAD, Hercules, CA, USA). Quantitative evaluation was performed using ImageJ software (1.48v).

### 4.8. Statistical Analysis

Unless otherwise specified, all experiments were repeated at least three times. Data were expressed as mean values with S.D. One-way ANOVA with Dunnett post hoc test and two-way ANOVA with Tukey’s multiple comparison test in GraphPad Prism software (8.0.1) were used for statistical analysis. Significance was set at *p* ≤ 0.05 (*), *p* ≤ 0.005 (**), *p* ≤ 0.0005 (***) and *p* ≤ 0.0001 (****).

## 5. Conclusions

Overall, our study demonstrated that combined treatment with metformin and S63845, in comparison to metformin and S63845 acting alone, had a stronger inhibitory effect on AML cell oxidative phosphorylation and glycolysis rate and consequently on cellular ATP levels. In addition, treatment-induced apoptotic cell death was concomitant with changes in levels of cellular ROS. Therefore, such modulation of cellular energetics and redox status might be beneficial in targeting chemoresistant AML. However, future studies are needed in order to verify these results in more clinically appropriate settings.

## Data Availability

The data presented in this study are available in Supplementary Material.

## Supplementary Materials

The following are available online. Table S1: Primers used for RT-qPCR analysis. Figure S1: Cell proliferation and viability assessment; Figure S2: Metformin and MCL-1 inhibitor S63845 induced changes in the expression of antioxidant system genes; Figure S3: Metformin and MCL-1 inhibitor S63845 effect on CDKN1A gene expression.

## Sample Availability

The data sets (data in native formats) used to support the findings of this study are available from the corresponding author upon request.
