# Supplementary material for: Pharmaceutical Drug Metformin and MCL1 Inhibitor S63845 Exhibit Anticancer Activity in Myeloid Leukemia Cells via Redox Remodeling
Source: Molecules. 2021 Apr 15;26(8):2303. doi: 10.3390/molecules26082303 (PMC8071510; doi:10.3390/molecules26082303)
Supplement: Supplementary file 1 [file molecules-26-02303-s001.zip › Appendix No 1.pdf]

Pharmaceutical drug metformin and MCL1 inhibitor S63845 exhibit anticancer activity in myeloid leukemia cells via redox remodeling

Giedrė Valiulienė[1*], Aida Vitkevičienė[1], Giedrė Skliutė[1], Veronika Borutinskaitė[1] and Rūta Navakauskienė[1]

[1]Department of Molecular Cell Biology, Institute of Biochemistry, Life Sciences Center, Vilnius University, Sauletekio av. 7, LT-01257 Vilnius, Lithuania

Table S1. Primers used for RT-qPCR analysis

Gene	Forward and reverse primers
BCL2	F: CGGAGGCTGGGATGCCTTTG<br>R: TTTGGGGCAGGCATGTTGAC
BCL2L1	F: TGCATTGTTCCCATAGAGTTCCA<br>R: CCTGAATGACCACCTAGAGCCTT
CAT1	F: TGGAGCTGGTAACCCAGTAGG<br>R: CCTTTGCCTTGGAGTATTTGGTA
CDKN1A	F: GGCAGACCAGCATGACAGATT<br>R: GCGGATTAGGGCTTCCTCT
GPX	F: CAGTCGGTGTATGCCTTCTCG<br>R: GAGGGACGCCACATTCTCG
HPRT1	F: TGGCGTCGTGATTAGTGATG<br>R: ACCCTTTCCAAATCCTCAGC
MCL1	F: GTGCCTTTGTGGCTAAACACT<br>R: AGTCCCGTTTTGTCCTTACGA
MYC	F: AATGAAAAGGCCCCCAAGGTAGTTATCC<br>R: GTCGTTTCCGCAACAAGTCCTCTTC
NRF1	F: AGGCTGGGGGAAAGAAAG<br>R: CCAACCTGGATAAGTGAGAC
TXN	F: TGAAGCAGATCGAGAGCAAGA<br>R: ACGTGGCTGAGAAGTCAACTA
TXN2	F: CGAGTGGTCAACAGTGAGACA<br>R: CCGCTGACACCTCATACTCA
TXNIP	F: TGTGTGAAGTTACTCGTGTCAAA<br>R: GCAGGTACTCCGAAGTCTGT
TXNRD1	F: ATATGGCAAGAAGGTGATGGTCC<br>R: GGGCTTGTCCTAACAAAGCTG
TXNRD2	F: CTAGCCCCGACACTCAGAAGA<br>R: GGCCATGATCGCTATGGGT

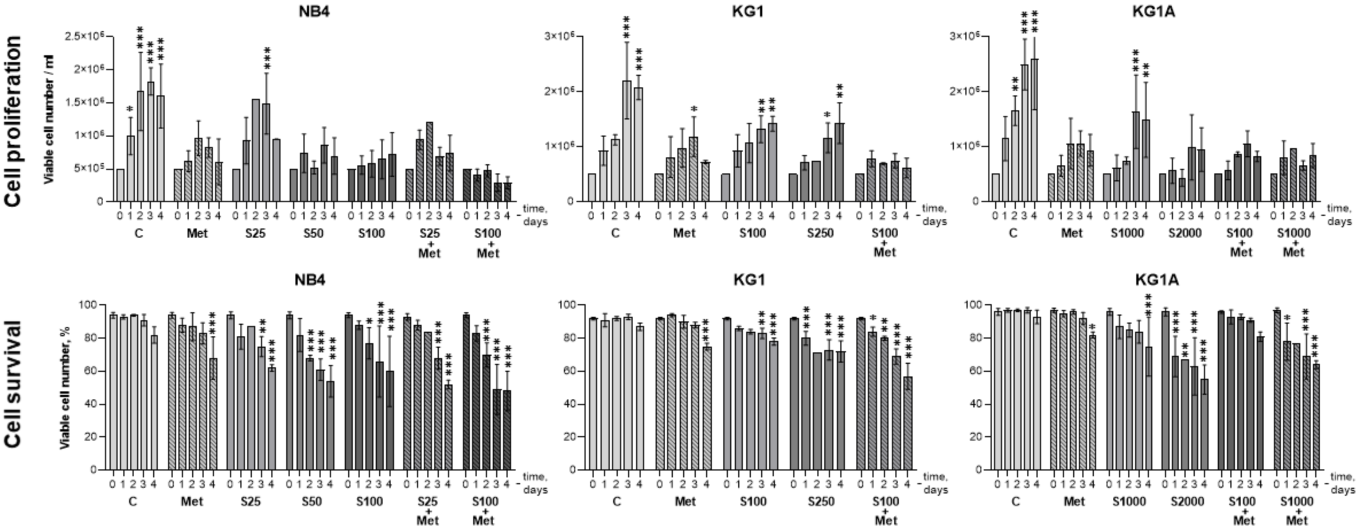

Figure S1. *Cell proliferation and viability assessment.*

NB4, KG1 and KG1A cells were treated with 10 mM metformin and with different concentrations of MCL-1 inhibitor S63845 (S25 – 25 nM of S63845, etc.). Cell proliferation and survival were evaluated by trypan blue exclusion test. Results are mean ± S.D. ($n \geq 3$). C – denotes control, untreated, cells. *Note: * denotes significant difference between treated vs. control cells with $p < 0.05$, ** denotes significant difference with $p < 0.01$ and *** denotes significant difference with $p < 0.005$, as evaluated using 1way ANOVA with Dunnett post-hoc test.*

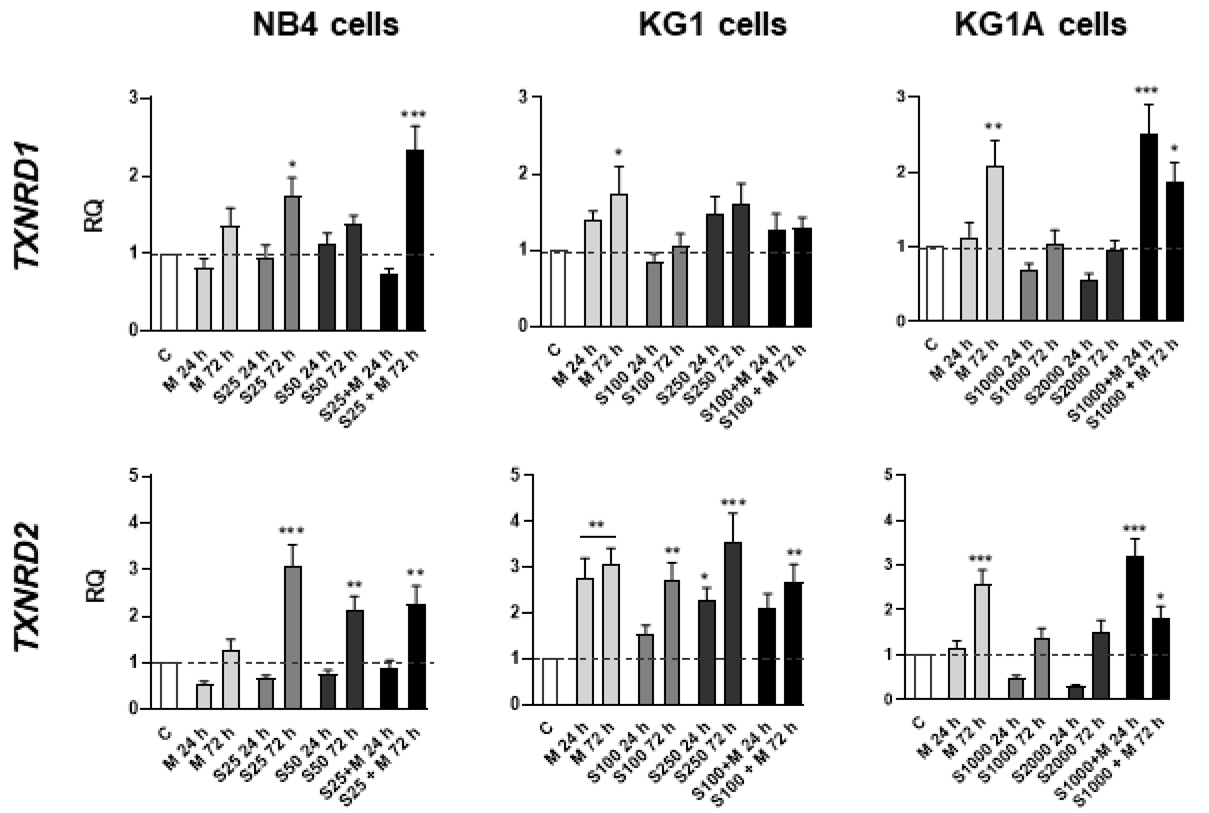

Figure S2. *Metformin and MCL-1 inhibitor S63845 induced changes in the expression of antioxidant system genes.* NB4 cells were treated with 25 nM and 50 nM S63845, 10 mM metformin and combination of 25 nM S63845 + 10 mM metformin. KG1 cells were treated with 100 nM and 250 nM S63845, 10 mM metformin and combination of 100 nM S63845 + 10 mM metformin. KG1A cells were treated with 1000 nM and 2000 nM S63845, 10 mM metformin and combination of 1000 nM S63845 + 10 mM metformin. Gene expression changes of antioxidant system genes *TXNRD1* and *TXNRD2* after 24 and 72 hours of treatment were measured using RT-qPCR ΔΔCt method. *HPRT1* gene expression was used for normalization; results are presented as relative changes in comparison to untreated cells; results are mean ± S.D. (n = 3). *Note: * denotes significant difference treated vs. control cells with* $p < 0.05$, *** denotes significant difference with* $p < 0.01$ *and *** denotes significant difference with* $p < 0.005$, *as evaluated using 1way ANOVA with Dunnett post-hoc test.*

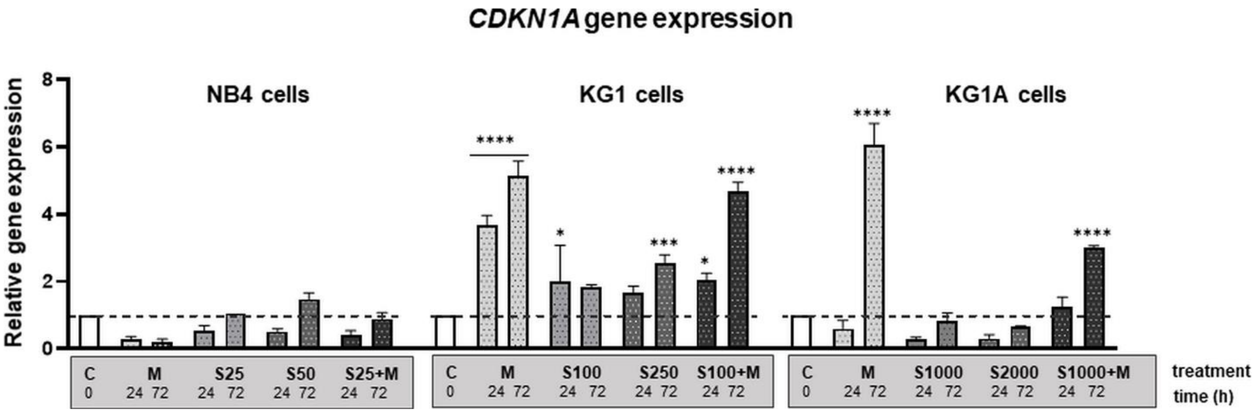

Figure S3. *Metformin and MCL-1 inhibitor S63845 effect on CDKN1A gene expression.*

Gene expression changes of Cyclin-dependent kinase inhibitor 1 (CDKN1A) after treatments were measured using RT-qPCR ΔΔCt method. HPRT1 gene expression was used for normalization; results are presented as relative changes in comparison to untreated cells; results are mean ± S.D. (n = 3). *Note: * denotes significant difference between treated vs. control cells with p <0.05, *** denotes significant difference with p < 0.005 and **** denotes significant difference with p < 0.001, as evaluated using 1way ANOVA with Dunnett post-hoc test.*
